# Prevalence of human immunodeficiency virus infection among transgender men in Rawalpindi (Pakistan)

**DOI:** 10.1186/1743-422X-9-229

**Published:** 2012-10-08

**Authors:** Hashaam Akhtar, Yasmeen Badshah, Samar Akhtar, Naghmana Kanwal, Maha Nadeem Akhtar, Najam us Sahar Sadaf Zaidi, Ishtiaq Qadri

**Affiliations:** 1 Atta-ur-Rahman School of Applied Biosciences, National University of Sciences and Technology, H-12 Sector, Islamabad, 44000, Pakistan; 2 Riphah Institute of Pharmaceutical Sciences, Riphah International University, 7th Avenue, Sector G-7/4, Islamabad, Pakistan

**Keywords:** Human immunodeficiency virus (HIV), Acquired immunodeficiency syndrome (AIDS), Eunuch, Transgender Pakistan, Sex rate

## Abstract

**Background:**

Transgender males are at high risk for sexually transmitted diseases including AIDS caused by the notorious Human Immunodeficiency Virus (HIV), yet little consideration is given by the policy makers, researchers and non-governmental organizations (NGOs) towards this sensitive issue in Pakistan.

**Methods:**

In this study, we have investigated the prevalence of HIV infection among 306 transgender males with a median age of 29 years (range 15–64 years) residing in Rawalpindi, Pakistan. Rapid HIV antibody-screening methods including the strip test and Enzyme Linked Immuno-absorbent tests were employed to detect HIV antibodies among the subjects. For further confirmation, Polymerase Chain Reaction (PCR) was carried out. Statistical analytical techniques utilized included logistic regression and chi-square.

**Results:**

HIV-1 was found to be the predominant viral subtype. PCR confirmed 21.6% (Confidence Interval 0.17-0.26) of the respondents were reported being HIV positive. 15.7% of the transgender men who shave at home and 13.7% of the transgender men who were educated below 5th grade were found to have HIV.

**Conclusion:**

This study shows a very high prevalence of HIV among transgender males. Unawareness among these individuals about the ramifications of this infection owes largely to lack of education. The spread rate is alarming and HIV epidemic is imminent if awareness is not widespread.

## Introduction

Annual estimates state that more than 40 million people are infected with HIV/AIDS globally with an alarming death rate of 3.1 million [1]. HIV spread is mostly via vertical and horizontal transference, contaminated blood and its products, and IDUs [2,3]. Risky sexual behavior predicts that HIV spread is possibly greater in homosexual individuals than the heterosexual population and even so, more attention should be given to the matter [3-5].

Pakistan’s first case of AIDS was recorded in 1987 in Lahore [6,7] and since then, the number has increased dramatically to approximately 100,000 among the country’s population, which according to the census of 2008 was 145 million [8]. UNAIDS currently characterizes Pakistan as a country of high risk and low-prevalence for HIV [9]. A mixed (cross-sectional) study was conducted in the two cities of Pakistan; Rawalpindi and Abbottabad in 2008 showing HIV rate to be 2.4% among the transgender men [10]. Another study in year 2005 defined that 7% of the male sex workers and 2% of the ‘hijras’ (transgender males) in Karachi were infected with HIV [11].

The current study primarily focuses on the transgender men (males by nature but appearing as women); including a few individuals whom had undergone physical transformations through sex reassignment surgery, use of hormones or silicon fillings. Nearly all of them express feminine gender. The transgender category- *hijras* under study are dancers by profession to the common eye but almost all of them are involved in sexual activities added with several socioeconomic conditions favorable for the spread of HIV in this class. These include; low literacy rate, poverty and a notable unemployment rate darkening the future prospects for not only their own class but for the nation as well. Another discussion on ignorance and lack of awareness and care is due to the negligence of protective measures during sexual activity.

## Methodology

### Data collection methods and sampling

A cross sectional study among transgender men was conducted in Rawalpindi (the twin city of the nation’s capital Islamabad) from November 2009 to June 2010. The total population of this city is approximately 4.41 million (census 2008) and the total area of this city is 286 sq Km. Transgenders usually live in groups governed by a leader known as the *Guru*. Different regions of Rawalpindi were selected for survey and a total of 306 transgender men were randomly included from different locations. All of the transgender men included in the study belong to the most populous and low income areas of Rawalpindi, where many reside together in a single apartment due to low socio-economic backgrounds.

### Data collection

The study design was presented to and approved by the Institutional Review Board (IRB) of the Atta-ur-Rahman School of Applied Biosciences, National University of Sciences and Technology (Additional file 1). In addition the board formally approved the questionnaire and consent form to be used in this study. In-depth interviews and discussions were conducted with the focused group and information was gathered through the aid of their superior commander-the ‘guru’ and a modified version of pretested questionnaire, an instrument which was approved by London School of Hygiene and Tropical Medicine (in the UK) [10]. A written consent was taken from each individual before they were interviewed pertaining to the manipulation of the information they provided us. A trained interviewer questioned the subjects about sexual behavior, needle or blade sharing, usage of condoms, frequency of intercourse, educational level, marital status, etc. The survey was completed voluntarily and respondents were advised of their right to decline participation at any time. Furthermore, the respondents were free to exercise their right, not to answer any question that they found uncomfortable. The questionnaires remained anonymous and were given codes to ensure anonymity of the respondents.

### Measures

#### Sexual activity

Respondents were asked about their sexual activities and their age at first sex. Whether they have ever had sexual intercourse (numerical criteria for answers: yes = 1, no = 0). This study is different from already conducted studies on HIV transmission, as the focused group is always involved in homosexual activities.

#### Condom use

Respondents were asked whether they or their partners used a condom during the last time they had sexual intercourse. The two response options were yes = 1, no = 0. A previous report shows that in Rawalpindi, the main mode of transmission of HIV is sexual intercourse and injectable drug users [10]. Keeping this in mind, the frequency of condom use among transgender men is a key determinant of exposure to sexually transmitted diseases and was thus included in the present study.

#### Shaving behavior

Respondents were asked whether they shave their body and face at home or how often they visit the barber shop. The response options were given values as Home = 1, Barber= 2 and Both=3. Other modes of bladeless shaving like threading and use of tweezers were included in home category.

#### Marital status

The question, ‘Are you married?’ measured this variable. The coding categories were Married = 1, Unmarried = 0. Few of the married respondents had kids, which verified their heterosexual activities.

#### Age

Asking subjects to respond to the question, ‘What is your age?’ measured this variable. Age was measured as a continuous variable and then converted into dichotomous variable as age ≤ 29 years =1 and age ≥ 30 years =2.

#### Education

The three grade levels were coded into a dummy variable (till 5th grade = 1; till 10th grade = 2; higher than 10th grade = 3).

#### Sex rate per week

A variable was created to note sex rate per week of the respondents and response categories were defined as 1–20 times =1, 21–40 times =2, more than 40 times =3.

### Sample collection/preparation

For the samples, 5 ml of venous blood was collected from each subject and was transferred into EDTA anti-coagulating tubes. From the collected sample, 1.5 ml was centrifuged for 10 min at 2400 rpm to separate serum from the whole blood. Sera of all patients were screened for HIV-1 and HIV-2 antibodies by microparticle enzymes immunoassay (AxSYM, Abbott Diagnostics, Wiesbaden, Germany). ELIZA positive samples were further confirmed by PCR.

HIV testing was anonymous, but according to the lab numbers, all of them received their reports linked to survey data.

### Inclusion criteria

All transgender men- *hijras* were included.

### Exclusion criteria

Female sex workers, FTMs (Female to Male), transgender females, gender queer and drag queen/kings were excluded from this study.

### Data analysis

The data was analyzed using SPSS (16.0 version) for Windows 7 [12]. Descriptive analyses were conducted by calculating the frequency distribution of HIV among transgender men and other study variables like education, shaving behavior, etc. To test for statistically significant associations between education, shaving behavior, sex-rate per week, marital status and prevalence of HIV, a series of chi-square analysis was performed. Multiple logit regression model was used to depict the relationship between the chances of spread of HIV among transgender men and its dependency on some other factors related to them. Five predictor variables were considered for this purpose.

• Age of the Respondent (transgender)

• Sex frequency in a week

• Education of transgender men

• Marital status

• Shaving behavior

Firstly, two (2) variables Age and Sex frequency in a week were considered into categorical variables because sex frequency seems to have some relation with the age, as it was higher in young transgender men. An average of the sex frequency was also taken in some middle-age respondents, since their sex frequency raises during different local festivals but stays elevated for few days only. Education of transgender men and shaving behavior are categorical variables and so were split in binary variables. This was done in such a way that if a variable has 4 categories then it was transformed in 4–1 binary variables e.g. shave variable has 3 categories; shave themselves, from barber and both so it was split into two binary variables i.e. home and barber. Hence the 3rd function cannot be introduced since an intercept is present in the model and last category in each variable is a reference category. Hierarchical technique was used to select the model in which researcher decides to include or exclude a variable by keeping in mind the theory and p-values of the variables.

## Results

**Figure 1:**
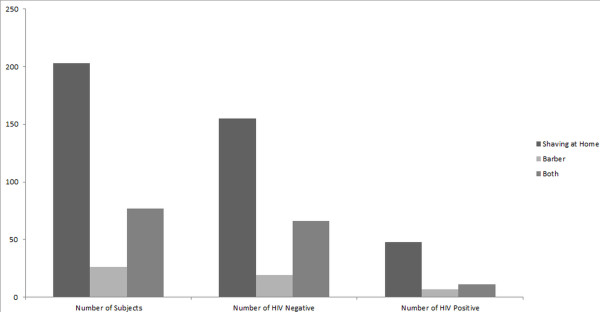
Distribution of the prevalence of HIV positive and negative patients according to their shaving habits; Shaving at home was much common in this class due to cultural norms and financial conditions but razor sharing was found to be a major issue.

Different parameters were considered for estimating the prevalence of HIV among transgender males, assummarized in Table 1. Overall HIV prevalence was found to be 21.6% in a total of 306 transgender men with 29 years as a median age (range 15–64 years) (Table 2). Results also reveal that 26.92% of the individuals who go to the barbers were HIV infected against 23.64% of those who shaved at home (Figure 1). Body shaving was found common among this class of sex workers and they commonly shared their razors due to lack of knowledge and shortage of assets.

**Table 1:** Cross tabulation of Age-Groups, education Level, shaving Behavior, sex-rate/week and marital status by prevalence of HIV among transgender men

**Variables**	**HIV negative n (%)**	**HIV positive n (%)**	**Prevalence (%)**	**P-value**
Age (in yrs)				
≤29	133 (43.5)	27 (8.8)	16.87	0.037
≥30	107 (35.0)	39 (12.7)	26.71	
Education				
till 5th Class	129 (42.2)	42 (13.7)	24.56	0.047
till 10th Class	72 (23.5)	21 (6.9)	22.58	
above 10th class	39 (12.7)	3 (1)	7.14	
Shave				
Home	155 (50.7)	48 (15.7)	23.64	0.185
Barber	19 (6.2)	7 (2.3)	26.92	
Both	66 (21.6)	11 (3.6)	14.28	
Sex rate/week				
1–20 times	170 (55.6)	35 (11.4)	17.07	0.012
21–40 times	47 (15.4)	24 (7.8)	33.80	
>40 times	23 (7.5)	7 (2.3)	23.33	
Marital Status				
Married	41 (13.4)	15 (4.9)	26.78	0.294
Unmarried	199 (65)	51 (16.7)	20.40	

**Table 2:** Sex starting ages and the acquisition of HIV

**Variables**	**Median age of total population**	**Median age of HIV positive transgender**	**Median age of HIV negative transgender**
Age (Years)	29	31	28
Sex Starting Age (Years)	15	14	15

**Figure 2:**
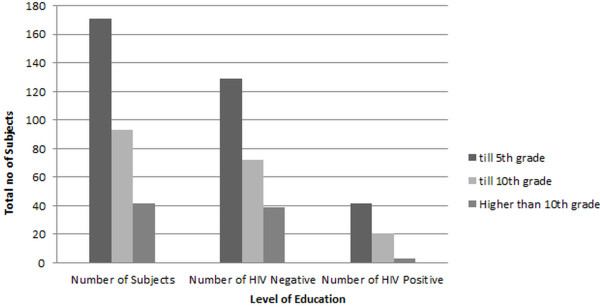
Number of HIV patients having different educational standards; Subjects with less than 5 years of education exhibited higher prevalence (24.56%) as compared to other groups.

Education was another factor that highly influenced the awareness and acquaintance of this mistreated class. This study (Figure 2) shows that HIV was least prevalent (7.14) in the most educated class (i.e. above 10th grade), whereas those who ran away from schools before primary (grade 5) were the most affected class. Looking at the age it is evident that it was also towards this age that they were sexually abused for the very first time.

**Figure 3:**
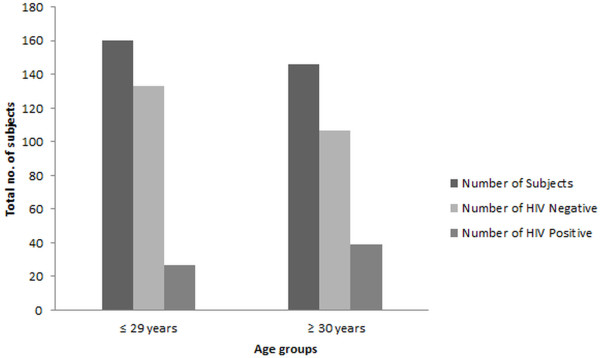
**Prevalence of HIV patients with respect to different age groups; Respondents within middle ages (20–40 years) were found to at higher risk, since the teenagers and those with 40-plus years of age were a minority of our study group. **The median age was found to be 29 years and more than 50% of HIV positive transgender males fall into middle aged group.

Transgender males with ages of 30 years and above were found to be at higher risk of having HIV as compared to those with an age of 29 or below although HIV was found prevalent in both age groups (Figure 3). Subjects above the age of 50 did not show HIV infection. During sampling process, it was found that most transgender males were of young age as many leave this occupation after 40 years of age and were therefore difficult to find. Owing to this 82.35% of the sampling group were transgender men of middle age (i.e. 20–39). However as depicted in Figure 3, the prevalence of HIV was higher in ≥ 30 year group.

Transgender men as dancers or beggars were found to have high prevalence of HIV, since they are easily accessible out in the open and also because their sex cost is low as compared to the dancers.

**Figure 4:**
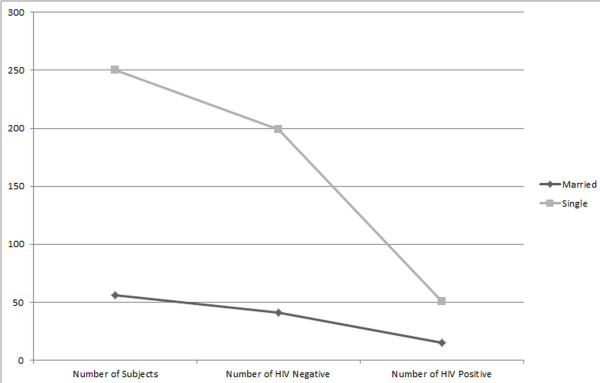
Number of heterosexual transgender males among the studied population; Graph clearly shows that quite a few heterosexual transgender males are at a risk of spreading HIV to the wives and children through horizontal and verticals routes respectively.

Another interesting factor disclosed in this study is about the heterosexual transgender men (HTM). Data obtained showed that 56 out of 306 respondents were married and the prevalence rate of HIV among these HTM was 26.78%. On the other hand, the prevalence rate of HIV in unmarried transgender men was 20.40% (Figure 4).

**Figure 5:**
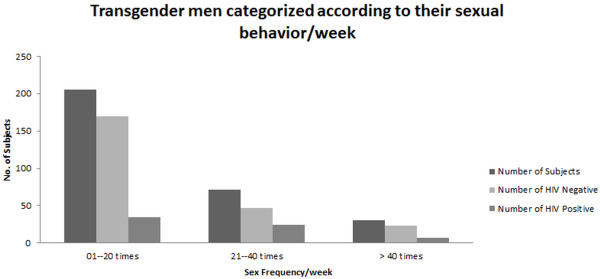
**HIV is dependent on sexual activities in transgender male; Transgender males who have sex rate of more than 20 per week are accelerating the spread of HIV although HIV is quite common in transgender males with less ratio of sex per week. **Respondents who were involved in sexual activities, more than 40 times per week also claimed inconsistency in reaching this number every week.

Finally it was also demonstrated that HIV infection depends on the sexual activities in addition to reuse of syringes and unscreened blood transfusions [13]. Hence same is the case with transgender males included in our study, where the prevalence increases with the increase frequency of sex per week (Figure 5). Not a single IDU was found during sampling so the flow of this virus mostly depends on the blade sharing and sexual behaviors.

Since hierarchical technique was used therefore sex frequency with a p-value of 0.012 was not found to be an important variable during modeling stage as 0.10 was used as a cut off point for p-value and concluded that the coefficient of the variable is not different from zero i.e. not a significant relationship with response variable. Two major reasons behind excluding this variable were that the reported per-sex-act probability of HIV transmission in a group of men, who have sex with men (MSM) is 0.82% per act for unprotected receptive anal intercourse, 0.06% for unprotected insertive anal intercourse and 0.04% for unprotected receptive oral intercourse with ejaculation [14,15]. Studies also reveals that 6000 times higher dose of virus is required to infect adult rhesus monkeys through oral exposure of simian immunodeficiency virus (SIV) (a virus closely related to HIV-1) than is required for rectal transmission [16]. Sensitivity of this question and the awkward answering pattern made this parameter unreliable in predicting the prevalence of HIV among transgender men.

The model shows that age, education, shaving habits and marital status have predictive power and the p-value of each coefficient can prove our claim. Furthermore it was found that the p-value of coefficient of marital status was greater than 0.10 (0.102) i.e. it does not have a significant prediction power but by excluding it, the other variable coefficients become insignificant. It can therefore be said that there might be a direct or indirect significant effect of this variable on HIV prevalence.

### Multivariate analysis

Logistic Regression was applied to the prevalence of HIV by educational level, shaving practices, age-group and marital status among transgender men and results from the logistic regression analyses are presented in Table 3.

**Table 3:** Odds ratios of Logistic regression of Educational level, shaving practices, age-group and marital status by prevalence of HIV among transgender men

**Variable**	**Coefficient**	**Standard error**	**P-value**	**Odds ratio**
Education				
till 5th grade	1.589	0.651	0.015	4.898
till matric	1.483	0.665	0.026	4.408
Shave				
Home	0.663	0.394	0.092	1.941
Barber	0.963	0.572	0.092	2.619
Age Group	−0.619	0.293	0.034	0.538
Marital Status	−0.623	0.380	0.102	0.537
Constant	−2.449	0.675	0.000	0.086

Table 3 shows the relative odds with respect to their daily routine or education level. During interviews, it was found that education was somehow related to awareness of different diseasesand their modes of transmission.

According to Table no. 3, the model can be written as

(1) $$\hat{p}=\frac{e^{-2.449-0.623Marital-0.619age+0.663home+0.963barber+1.483Matric+1.5895thgrade}}{1+e^{-2.449-0.623Marital-0.619age+0.663home+0.963barber+1.483Matric+1.5895thgrade}}$$

The above equation can be illustrated as follows

• $\hat{p}$ is the probability of prevalence of HIV

• Marital is marital status of respondent ( 1 for unmarried, 0 otherwise)

• home:shave at home (1 for shave at home, 0 otherwise)

• barber: shave from barber (1 for shave from barber, 0 otherwise)

• Matric: level of education is till matric (1 for education till 10th grade, 0 otherwise)

• 5th grade: education till or below 5th class. (1 for education till 5^th^grade, 0 otherwise)

• Age : age of the respondent taken as more then 29 (1 for age 29 and less 0 otherwise)

The analyses shows that all the variants have significant predicting power and we can see that a transgender men with education level till 5th class or lower, has 5 times greater odds than for a person with higher education level. Transgender men with education level between 5th and 10th grades have 4 times greater chances, than the odds for other levels of education of HIV prevalence.

Age was divided into two categories, i.e. ≤ 29 years and ≥ 30 years and it was found that the transgender men with ≤ 29 years have odds half than transgender men with age ≥ 30 years or in other words the transgender men with age ≥ 30 years have odds double than the transgender having age ≤ 29 years.

Respondents were also categorized into three groups according to their shaving behavior, i.e. shave at home, shave from barber or both. The third category was used as a reference in this model. Results show that the odds for shaving at home is two times greater than the other group and shaving from barber is almost two and half times more than match-ups. So keeping this in mind shaving is not that significant and shows weak linkages for prediction when categorized in different groups. So we can conclude similar results that shaving from barber is also risky in dispersing HIV [17]. Shave at home was also important in many cases though less odds as compared to barber visits but body shaving and blade sharing is a common practice among different transgender men, living together in a single apartment. Keeping the non-significance of the coefficient in mind, that can affect the results indirectly; we also concluded that the transgender men who were not involved in heterosexual activities had half odds of having HIV than the married ones.

## Discussion

Pakistan lacks documented data on the prevalence of HIV in the transgender community at the national level. Smaller regional studies, however, indicate that the transgender community has been one of the communities hit hardest by this epidemic. Transgender individuals face discrimination in a wide range of public and private settings, including employment, housing, health care, and access to social services. In Pakistan and few other Asian countries, stigma and discrimination against transgender individuals exacerbates their HIV risk, increasing the likelihood for substance abuse and survival sex and decreasing the likelihood of safer sex practices [2,18,19]. Among the factors that may place transgender men at increased risk for HIV in Asian countries are mental health concerns, physical abuse, social isolation, economic marginalization, incarceration, and unmet transgender-specific health care needs—all of which are heightened by stigma [2]. There is broad diversity among trans-communities including variations in sexual orientation and HIV risk in addition to racial and ethnic diversity. The terms “transgender” or “transsexual” do not imply any specific sexual orientation and transgender individuals identify across the full spectrum of orientations with some considering conventional labels inadequate or inapplicable to them.

In the present study, various behavioral parameters were taken into account to elaborate and discuss the prevalence of HIV in our study categorically. During the interviews, respondents were questioned about their occupations. Hardly any of the subjects admitted to having sex as their occupation, being a taboo topic. However on persuasion, the *Guru* (boss) revealed that all of them were involved in promiscuity regardless of what their *claimed* occupation was. According to PCR results highest prevalence was found in the middle-aged transgender men and most of them claimed to be dancers which can be explained by the fact that they have larger business owing to greater exposure, and recognition. Furthermore, it was elucidated that the educated lot of the *hijras* is cautious and aware about unsafe sex and its consequences. Therefore, they take more care by avoiding unsafe sex and resultantly have lower prevalence of HIV amongst them. Interestingly, young people who drop out of school are more susceptible to become sexually active at younger ages and thus according to this study many of the transgender males were first sexually abused in their teenage [20]. Lower literacy rate in this case was found to be directly proportional to the lack of awareness, which has miserably transformed the situation into a disaster.

Our data’s statistical analysis reveals that the *hijras* who visit barbers have potentially high risk of spreading or acquiring HIV as they face greater vulnerability to non-preventive measures while shaving i.e., blade re-use, no disinfection of instruments and frequent body shaves,etc [17]. Although, shaving at home was also not as safe as previously perceived. Body shaving is a regular exercise for most of them and they share razors in this activity, so an elevated prevalence could be expected in future, if ignoranceof the consequences persists. Most of the respondents had no awareness about the mode of transmission of viral diseases and had never been tested for them.

This study shows that heterosexual transgender males, are a greater threat of HIV spread than regular homosexuals. 18.3% of respondents were married to females and even had children: their marital status has no effect on their sexual activity and thus channel out a way for HIV to be transmitted into the general population, both vertically and horizontally. Nevertheless, majority of subjects live together as a clan. Only four individuals out of a total of 306 interviewed live with their families while many had hidden contacts with at least one member of the family unit.

Transgender males ranging 25–40 years of age, having only five years of professional experience were seen to be at eminent risk of acquiring HIV infection in relevance to other activities.A fraction of the subjects stated to have had entered the field because they had been sexually abused and astonishingly they built one by tenth of this *hijra* community. However, the majority turned to the *hijra* clan for acceptance, security, satisfaction of sexual desires and easy-money.

Injecting drug use is reportedly one of the major factors behind HIV transmission besides sexual contact. However this case study showed negligible injectable drug users (IDUs) thereby eliminating the risk of HIV spread via unsafe injections, needle sharing etc [21]. There were some alcohol addicted dancers amongst the respondents but their intake was relatively lower to be considered as a critical factor. Lastly the use of condom could also not be related or analyzed in this study because not even a single respondent accepted that he uses a condom with his customers or friends during sex. The reason for not using condoms came out to be an apprehension on the customer’s part and thus affecting their business.

The reported data clearly represents the problems in our population. The problems are further complicated by social stigmas of cultural and religious origin in Pakistan. Furthermore, building an HIV prevention response that effectively addresses the needs of transgender communities requires the active participation of the communities along with service providers, local public health leadership, and community planning or advisory groups. Above all, it is important that healthcare providers get to know the specific context and living situation of transgender populations from the regions in which they are working. Beyond the general fact that transgender people live partially or completely in a sex or gender that was not assigned to them at birth, they are extremely diverse. Their specific HIV‐related issues and challenges depend on the particular social determinants of health that affect them in their region, but also on the way they see themselves and on the body modifications they seek out.

## Conclusion

The prediction of HIV and further STI epidemic in Pakistan is now proximate. Precautionary measures should be taken now or it will be too late since the rate of spread in Pakistan is much higher than any of the western countries [10]. Having gone through the results of the study conducted regarding the occurrence of HIV in Transgender males or *hijras*inin a major city –Rawalpindi, adjoining Pakistan’s capital, a number of issues concerning HIV control come to light. Illiteracy, ignorance and background status explain the amateurish behavior. Besides that, the transgender class is looked down upon and secluded from the society as they never were accepted as normal. Awareness, protective behavioral education and health care services should be available and in approach of this community.

Our survey is primarily based on the data collected through interviews thereby there is no certainty behind the risk prevention measures of the respondents. The particularity of this case study is limited to the city of Rawalpindi only.

Furthermore, the transgender class being dealt with focuses only on transgender men because the MTFs have an infection rate four times greater than that of female sex workers [22].

Interventions and programs should recognize and reflect all of these differences, including racial and ethnic issues that contribute to HIV risk. This is especially important when adapting interventions originally designed for other populations. HIV prevention programs are often a gateway for transgender people into social service or health care systems. For many transgender individuals, these programs are the first place where they feel safe and respected. In approaching HIV prevention for transgender men, it is important to remember that many of their basic survival needs, including health care, may be unmet as a result of stigma and discrimination and HIV/AIDS prevention may not be a high priority.

### Implications of the study

The findings and the conclusions of this study have implications for health care practitioners, NGO’s and health educators trying to better understand the prevalence of sexual activity and risk behaviors among transgender men in Rawalpindi and perhaps other cities of Indo-Pak. The prevalence of HIV, the sexual activities and the use of condom among transgender men of the current study suggests that current educational efforts, awareness programs and strategies are inadequate. The conclusion of this study suggests that transgender men should be provided with proper medical facilities and treatments after screening, furthermore discerning the dispersing pattern of HIV was also an important aspect of this study.

### Declared ethical approval

All the samples were collected, with the approval of the Institutional Review Board, Atta-ur-Rahman School of Applied Biosciences, National University of Sciences and Technology (NUST), Islamabad, Pakistan. The candidates who gave permission were informed about the tests to be conducted from that blood samples, furthermore the interviews were conducted in front of the *Guru* (chief or supervisor) of that community.

## Competing interests

The authors declare that they have no competing interests.

## Authors’ contributions

HA and SA carried out sampling, participated in the statistical analysis and drafted the manuscript. YB carried out the Molecular lab testing. HA, NK and MNA participated in the design of the study and performed the statistical analysis. NSSZ and IQ conceived of the study, and participated in its design and coordination. All authors read and approved the final manuscript.

## Supplementary Material

Additional file 1 **Performa for interviewing transgender males. **Form used for collecting information about transgender males. Click here for file
