# Supplementary material for: Prevalence of human immunodeficiency virus infection among transgender men in Rawalpindi (Pakistan)
Source: Virol J. 2012 Oct 8;9:229. doi: 10.1186/1743-422X-9-229 (PMC3558455; doi:10.1186/1743-422X-9-229)
Supplement: Additional file 1 — Performa for interviewing transgender males. Form used for collecting information about transgender males. [file 1743-422X-9-229-S1.pdf]

NUST CENTER OF VIROLOGY AND IMMUNOLOGY (NCVI)

All Data can only be used by HASHAAM AKHTAR (all rights Reserved)

Date: - : : 2009

Name of person: ____________________________

Age: ________

Sexual Activities : ( ) YES ( ) NO
Duration : _______ Years / months
Started at age: _____ Years / months
Test of Sex Partner: ( ) YES ( ) NO
Use Of Condom: ( ) YES ( ) NO
Gender: ( ) ( )

Disease: ______ ______ ______

Duration of disease: ________________

Treatment:

( ) Homeo ( ) Herbal ( ) Allopathy ( ) Spiritual

Diabetes test :______mg/dL
Reference : ______mg/dL

Body temperature: _____ F
Reference: up to

Blood Pressure: ______mmHg
Reference: 120/80 mmHg

Pulse Rate: ______ /min
Reference 72/min

Height: _____ cm
Waist: _____ cm
Weight: _____ kg

Name of medicine:

________________________ __________________________
________________________ __________________________
________________________ __________________________
________________________ __________________________

Any Skin disease or Dandruff : ____
Any type of Fatigue or body pain: ____________________________________
Any Tooth related problem: ______ **(Dentist Category ( ) Street ( ) hospital)**
Name and genotyping of virus or bacteria: _______________________________
Any vaccination : _____________ ________________ _____________ ________
Shave : ( ) Home ( ) Barber ( ) Both
Sexual Life : ( ) Yes ( ) No ( ) ____
Type of Drug Abused: ( ) inj ( ) Tablet ( ) cigarette ( ) Naswar ( ) Hashish ( ) Marijuana
Name of Drug used: ____
____
Phone no: ________________________ ______________________________
Address: ____
__________________________________ **City**_____________________________
Current status: ____
( ) Strip test for HCV ( ) Strip test for HBV ( ) Other ________
Interferon Resistance:-
After (___injs)
Any other Disease ( ) T.B ( ) Diabetes ( ) B.P ( ) ________
